# Inflammation and Neuro-Immune Dysregulations in Autism Spectrum Disorders

**DOI:** 10.3390/ph11020056

**Published:** 2018-06-04

**Authors:** Dario Siniscalco, Stephen Schultz, Anna Lisa Brigida, Nicola Antonucci

**Affiliations:** 1 Department of Experimental Medicine, University of Campania, 80138 Naples, Italy; 2 Italian Group for Study Autism—GISA, 25018 Brescia, Italy; brigida.annalisa@gmail.com; 3 Centre for Autism—La Forza del Silenzio, 81036 Caserta, Italy; 4 Department of Cellular and Integrative Physiology, School of Medicine, University of Texas Health Science Center San Antonio, San Antonio, TX 78229, USA; stevendri0629@gmail.com; 5 Biomedical Centre for Autism Research and Treatment, 70124 Bari, Italy; info@antonucci.eu

**Keywords:** autism, monocytes, macrophages, inflammation, neuro-immune system

## Abstract

Autism Spectrum Disorder (ASD) is characterized by persistent deficits in social communication and interaction and restricted-repetitive patterns of behavior, interests, or activities. Strong inflammation states are associated with ASD. This inflammatory condition is often linked to immune system dysfunction. Several cell types are enrolled to trigger and sustain these processes. Neuro-inflammation and neuro-immune abnormalities have now been established in ASD as key factors in its development and maintenance. In this review, we will explore inflammatory conditions, dysfunctions in neuro-immune cross-talk, and immune system treatments in ASD management.

## 1. Autism Spectrum Disorder (ASD)

Autism Spectrum Disorder (ASD) is defined by the Diagnostic and Treatment Manual for Mental Disorders, Fifth Edition (DSM-5) and is characterized by persistent deficits in social communication interaction and restricted-repetitive patterns of behavior, interests, or activities. These symptoms begin in early childhood, and produce clinically significant developmental impairment [1]. The DSM-5 combined the previously separate subtypes of ASD listed in the Diagnostic and Statistical Manual for Mental Disorders, Fourth Edition (DSM-4). Autistic disorder, Asperger syndrome, pervasive developmental disorder-not otherwise specified (PDD-NOS), and childhood disintegrative disorder are now combined into one diagnosis of ASD. Two of the prominent clinical features of ASD are inflammation and neuro-immune system dysregulation [2,3,4].

In a 2013 review article, we summarized environmental factors which could contribute to ASD pathogenesis through epigenetic modifications [5]. Since the publication of that review, additional articles have continued to add to the evidence of epigenetic modifications in ASD. Some of these epigenetic modifications include DNA methylation, epigenetic proteins, gene polymorphisms associated with variation in diet, histone modifications, and microRNA (miRNA) dysregulation [6,7,8,9,10]. Of note, changes in gene expression in individuals with ASD indicate alterations in common molecular pathways, including immune system pathways, could contribute to ASD [9].

## 2. Inflammatory Conditions in Autism Spectrum Disorders

In the recent years, studies increasingly indicate a strong inflammatory state associated with ASD [11]. This inflammatory condition is often linked to immune system dysfunction [12]. Enhanced inflammatory activity in ASD children has been demonstrated through pro-inflammatory biomarkers analysis. Interleukins are signalling proteins, belonging to the cytokine family, responsible for immune modulation and inflammatory responses [13]. These proteins are optimal biomarkers for studying inflammatory states. In a milestone paper, pro-inflammatory cytokines were found to be elevated in plasma of 97 medication-free and healthy children (2 to 5 years old) with ASD, recruited in the population based case-control Childhood Autism Risks from Genetics and Environment (CHARGE) study, compared with age-matched typically developing control children and children with other developmental disabilities [14]. Increased cytokine levels, IL-1β, IL-6, IL-8 and IL-12p40 among others, were associated with impairments in stereotypypical behaviors and in regression, suggesting that dysfunctional immune responses could affect core behaviors in ASD [14]. Over-production of pro-inflammatory cytokines was also demonstrated in vitro by utilizing cultured and stimulated peripheral blood monocytes from ASD children [15]. Increased pro-inflammatory Th-2 cytokines were found, not only in plasma, but also in peripheral blood mononuclear cells of twenty ASD children (from 3 to 10 years old) compared to age-matched normally developing controls [16]. Thirteen ASD children were under psychotropic drug treatments, however no significant differences were observed for any cytokines examined. As a limitation, the authors indicated that some psychotropic drugs used for ASD symptoms, such as lithium, benzodiazepines, and the atypical antipsychotic clozapine, could be able to increase cytokine levels [16]. Furthermore, both Th1 and Th2 cytokines have been reported to be increased in ASD children [16], and most recently, plasma levels of IL-1β, IL-6, IL-17, IL-12p40, and IL-12p70 cytokines were found elevated in seventeen children (3–9 years old) with clinical diagnosis of ASD as compared to fifteen age-matched normally developing children [17]. Interestingly, plasma cytokine profiles were different between the two grades of severity in which ASD children have been subtyped (moderate and mild severity) according to the Childhood Autism Rating Scale (CARS) test: higher levels of IL-12p40 in patients with mild disease severity and higher levels of tumor necrosis factor alpha (TNF-α) in patients with moderate severity [17]. TNF-α in sera of 32 ASD children was found to be over-produced in another study [18]; also in this further case, TNF-α levels were positively correlated with the ASD severity (as tested by Autism Behavior Checklist, ABC), likely indicating an ASD phenotype. ASD children showed decreased expression of the TNF-α and hnRNPL-related immunoregulatory LincRNA (THRIL) gene involved in regulating TNF-α [18]. Plasma cytokine levels have been shown to be altered in 87 Chinese ASD children (2–6 years old). In this study, eotaxin, TGF-b and TNF-α were up-regulated while no differences were observed in IL-8 levels between ASD (assessed using the Autism Diagnostic Observation Schedule, second edition, ADOS-2) and 41 normally developing age-matched control children [19]. Interestingly, in this study, most of the recruited ASD children were between the moderate and severe end of the ADOS-2 scale. It would be interesting to analyze cytokine levels in ASD children with mild symptoms. An Iranian-based study has recently analyzed the whole blood mRNA expression levels of cytokines of 30 ASD patients and 41 age- and sex-matched healthy subjects by real-time PCR. In ASD children, TNF-α, IL-6 and IL-17 were significantly up-regulated, whereas IL-2 expression was down-regulated [20]. Expression profiles of the examined cytokines showed a sex-specific correlation. TNF-α mRNA expression was correlated with the mRNA expression of TGF-β, IFN-γ, IL-17 and IL-6 in male subjects, but none of these correlated with TNF-α in female subjects [20]. Increased peripheral concentrations of S100B and TNF-α were found in plasma of 40 unmedicated ASD children compared to 35 healthy normally developing controls [21]. No differences in IL-1β, IL-6 and IFN-γ were noted. By way of explanation of this incongruence with other studies, the authors reported that Autism Diagnostic Interview Revised (ADI-R) and the Autism Diagnostic Observation Schedule (ADOS) were not used in their country [21]. Over-production of TNF-α, IL-1 and IL-6 from the sera of seventy-seven ASD, attention deficit disorder, Rett’s syndrome, and Asperger syndrome children has been demonstrated in central Saudi Arabia [22]. Social withdrawal, sleeping, and eating disorders were found among ASD subjects compared to age and sex matched control children [22]. Furthermore, increased pro-inflammatory cytokine levels were found in plasma of a small sample size of 48 unmedicated high-functioning ASD subjects compared to matched control subjects [23]. Cytokine profiles in the above mentioned referenced articles are summarized in Table 1 and Table 2.

Several hypotheses have been offered to explain ASD-related inflammation. Dysregulation of maternal immune system during pregnancy has been implicated in the development of ASD [24]. The transfer of maternal fetal brain-reactive antibodies is associated with increased risk of ASD [25]. In maternal autoimmunity, IgG antibodies derived from the mother enter the fetal compartment by freely crossing the placenta; these antibodies recognize self-proteins and can interfere with fetal development. In addition, the blood brain barrier is not completely formed in the fetus. IgG reactivity against fetal brain proteins was found in plasma from 7 of 61 mothers (11.5%) of ASD children, but not in 62 mothers of normally developing children. Protein reactivity was toward fetal, non-adult, brain [26]. A larger study correlated the brain-specific autoantibodies and behavior in 277 ASD children and 189 typically developing age-matched controls. Children with the presence of autoantibodies showed lower adaptive and cognitive function and increased aberrant behaviors [27]. It has been proposed that maternal autoantibodies could be used as useful markers for ASD diagnosis [28], at least for a specific ASD endophenotype [29].

The gastrointestinal system has a direct connection with the immune system. ASD-associated gastrointestinal symptoms could be a manifestation of an underlying inflammatory process [30]. Increased intestinal permeability has been associated with ASD (leaky gut hypothesis) [31]. A milestone paper detected, by means of lactulose/mannitol test, increased intestinal permeability in 37% of autistic patients and in 21% of their relatives. Interestingly, autistic subjects on gluten-casein-free diet had significantly lower intestinal permeability than the values of those who were on an unrestricted diet and control subjects. Calprotectin was used as non-invasive marker to assess intestinal inflammation [32]. Intestinal barrier defects predispose ASD patients to be sensitized by environment antigens. Of note, the abnormal immune system in autistic subjects could be due to gluten/casein derived molecules that, once moved to a damaged intestinal barrier, trigger pro-inflammatory processes (increased pro-inflammatory cytokines and pro-inflammatory monocytes) [33]. These pro-inflammatory mediators or immune-activated complexes reach the higher brain centers through the bloodstream, and a permissive blood-brain barrier further collaborates in neuro-inflammation events [34].

Increased evidence supports gut microbiota dysbiosis in ASD [35]. By means of culturing methods, aggressive forms of *Candida* spp. have been identified in the stool of 57% of ASD children and not in age-matched healthy controls [36]. Decreases in *Lactobacillus* spp. and *Clostridium* spp. could sustain the dysbiosis. Different fecal flora have been observed in regressive autism compared to normal controls [37]. Harmful gut-colonizing microorganisms produce several chemicals from their cell cycles that affect behaviors, as these metabolites show similar molecular structures to brain neurotransmitters [38].

## 3. Dysfunctions in Neuro-Immune Cross-Talk in Autism Spectrum Disorder

The inflammatory state associated with ASD is also reflected in the central nervous system as brain inflammation [39,40]. Human post-mortem samples, as well as animal models, suggest increased levels of IL-6 in autistic brain, where this cytokine is able to mediate neuro-anatomical abnormalities [41,42]. Indeed, excitatory and inhibitory synaptic formations and transmissions are altered by IL-6 over-production, as well as the shape, length and distribution pattern of dendritic spines [43]. Several brain areas of autistic patients show signs of active neuro-inflammatory process: cerebral cortex, white matter, and cerebellum [44]. Neuroglia responses are activated toward pro-inflammatory processes involving astroglia and microglia [44]. Remarkably, it has been demonstrated that there is increased expression IL-1β, IL-6, IL-17 and TNF-α in the autistic brain [45]. Neuro-inflammation, driven by increased production of pro-inflammatory cytokines, could be the principal mechanism in the pathophysiology of ASD [46,47]. Of course, systemic inflammation, up-regulation of IL-1β, IL-6, IL-17, IL-18, IL-33, TNF-α, and glia/microglia activation have also been demonstrated in animal models of ASD [48]. 

Taken together, all these studies indicate peripheral and central inflammatory responses with dysregulation of specific key cytokines in ASD. Cytokine profiles in above mentioned referenced articles are summarized in Table 3.

TNF-α, IL-1β, IL-6, and IL-17 are the pro-inflammatory molecules most involved and up-regulated. Of note, TNF-α, mainly produced by mast cells and activated monocytes/macrophages, is able to regulate immune cells through IL-6 production and, in turn, is under the control of IL-1β [49,50,51]. ASD monocytes show strong impairment and altered immune responses. An imbalance has been demonstrated in the production of pro-inflammatory IL-1β and IL-6 cytokines and anti-inflammatory IL-10 cytokine by monocytes from ASD patients, which reflect changes in behavioral symptoms in the ASD subjects [52]. Monocytes are blood mononuclear cells which are extremely important in the immune system. They are the precursors of macrophages and dendritic cells (after chemotaxic migration into tissues) and key regulators of the immune responses, as they contribute to the local chemical micro-environment by cytokine production and release, phagocytosis, and antigen presentation. Aberrant responses of monocytic cells could trigger long-term immune alterations in ASD [12]. Molecular systems are dysregulated in ASD-monocytic cells. Transcription profiling of monocytes in ASD children revealed over a 2-fold up and down regulation of genes, as compared to normal controls. Gene expressions of TGFbR-, Notch- and EGFR1-related pathways were enriched in the ASD monocytes [53]. The caspase pathway was found to be aberrantly dysregulated in ASD monocytes [54]. Cysteinyl aspartate-specific proteases (caspases) are enzymatic proteins involved in the regulatory and execution phases of programmed cell death (apoptosis) [55]. Beyond apoptosis, caspases control several other processes inside the cell, such as inflammatory, innate, and adaptive immune responses, and also differentiation of monocytes into macrophages [56,57]. Very interestingly, in an in vitro model, ASD blood monocyte-derived macrophages show strong impairments in the endocannabinoid system [58]. This intricate network of lipid-derived signalling molecules has been already been demonstrated to be altered in ASD monocytes [59] and could represent the molecular link between the inflammatory state and neuro-immune alterations of ASD [12].

In ASD monocytes, caspase-1 gene was over-expressed [54]. Caspase-1 catalyzes the hydrolysis and activation on the pro-inflammatory cytokines IL-1β and IL-18, further contributing to long-term immune alterations. Most recently, ASD-monocytic cells show enhanced activation of IL-17 receptor signalling, aggravating neuro-inflammation in ASD [60]. Finally, ASD monocytes are committed versus a pro-inflammatory state [61]. This condition, together with over-production of pro-inflammatory cytokines, could be reflected in higher brain areas of ASD subjects. Indeed, in ASD, blood brain barrier integrity is disrupted with an increase of neuro-inflammatory processes [34,62]; the increased permeability of the barrier membrane allows free entrance of pro-inflammatory signalling molecules released by circulating monocytes, modulating neural function. Several neural/immune cell types are involved. Mast cells are induced to release IL-6 and TNF, causing focal brain inflammation [63]. Microglia, in turn, are stimulated to activation and proliferation, leading to disruption of neuronal plasticity [62,64,65], and in this way trigger social interaction, communication, and behavior impairments [66]. Microglia are derived from the monocyte/macrophage lineage and are considered the primary immune cells or resident macrophages in the CNS [67]. Indeed, in psychiatric diseases, microglia are committed versus the M1 pro-inflammatory state, resulting in an imbalance of M1/M2 (anti-inflammatory) phenotypes [68]. Immunohistochemical analysis of brain slices (cerebellum, midfrontal, and cingulate gyrus) from autopsy in 18 ASD patients indicated marked activation of microglia and astroglia, with strong over-production of neuroglia-derived macrophage chemoattractant protein (MCP)-1 and TGF-β1, indicating an active neuro-inflammatory process in the cerebral cortex, white matter and cerebellum [44]. Dysregulated microglial responses co-expressed with altered neuronal activity-dependent genes in ASD brains were also detected by transcriptome analysis [69,70]. Allergic responses co-morbid with ASD are associated with M2A-polarized macrophages and microglia [71]. These cells are able to readily shift from immature forms and among several M substrates depending on the local chemical microenvironment signalling [58]. M2A phenotypic state is referred as alternative activation and is driven by IL-4 and IL-13 cytokines towards reparative/recovery functions [72]. Alterations in macrophages/microglia responses are particularly harmful in ASD. Very recently, it has been demonstrated that these cells are induced by inflammatory stimuli toward an acute immune training and tolerance, through epigenetic reprogramming, for at least six months. Given this, the immunological imprinting could enhance and sustain inflammation for a long time [73].

A key role for vitamin D in autism development has been discovered. Vitamin D, through its ligand-activated nuclear hormone receptor (VDR), regulates the expression of pro-inflammatory genes and plays an immunoregulatory role [58]. Monocytes, macrophages, and lymphocytes express VDR on the cell surface. It has been demonstrated that genetic polymorphisms of VRD, influencing vitamin D3 (cholecalciferol) metabolism, could correlate with ASD development [74].

Among immune system dysfunctions implicated in ASD, reduced natural killer (NK) cell activity has been demonstrated [75]. Of note, the decrease in NK cell function could be the result of increased production and release of nitric oxide (NO) by activated microglia [76,77]. CD57-positive NK cell dysfunction has also recently been demonstrated in a large cohort of 104 ASD subjects compared to age-matched healthy controls [78], further corroborating the changes in ASD neuro-immune cross talk. NK cells drive host defence processes against infections; abnormal NK cell-mediated responses could predispose autistic children to infections [79]. ASD subjects with immune homeostasis deregulation are more susceptible to various infections, including active/aggressive forms of *Candida* spp. and *Clostridium* spp. [36].

In the CNS of autistic patients, astrocytes are also involved in ASD development [80]. These cells are able to modulate several key events in synaptic processes: neurotransmitter homeostasis, recycling of substrates, synaptogenesis and synaptic remodelling, activity, and plasticity. Astroglia could be involved in ASD through signalling of metabotropic glutamate receptor subtype-5 (mGluR5). The G-protein coupled receptors are a class of receptors involved in ASD and modulation of mGluR5 is effective in animal models of neuro-degeneration, inflammation and ASD [81,82,83,84]. 

Using transcranial ultrasonography, it has been reported that ASD children show abnormally increased extra-axial fluid (EAF) [85]. This event could be linked to inflammatory changes due to stagnation of the cerebrospinal fluid (CSF) flow and to prolonged exposure to toxic chemical messengers of inflammation. Increased expression of neuro-inflammatory markers in CSF have a role in ASD development [86].

Other important immune cell subsets show abnormalities in ASD. By means of flow cytometry, CD8(+) B and T lymphocyte numbers were increased in 59 adult ASD subjects compared to 26 control adults [87]. Alterations in activation profiles for T cells and in adaptive cellular immune functions have been demonstrated in 66 children (2–5 years old) with ASD compared to 73 age-matched typically developing controls [88]. Counts of CD3(+), CD4(+) and CD8(+) T cells expressing activation markers CD134 and CD25 but not CD69, HLA-DR or CD137 were significantly reduced in the ASD population, indicating an altered activation profile for T cells. CD19(+) B lymphocytes are increased and CD4(+) T helper cells were decreased in 45 ASD children and teens (3–15 years old) [89]. As an ASD model, maternal immune activation (MIA) mouse offspring display altered immune functions with systemic deficits in CD4(+) TCRβ(+) Foxp3(+) CD25(+) T regulatory cells and increased IL-6 and IL-17 cytokine production by CD4(+) T cells [90]. Central neuro-inflammation and altered inflammatory responses, together with synaptic alterations, have been demonstrated in well-characterized valproic acid (VPA) rodent models of autism [91,92].

## 4. Immune Treatments in ASD Management

The immunodeficiency and the autoimmunity in ASD patients have been proposed as the rationale for the use of intravenous immunoglobulin (IVIG) infusion as a therapeutic tool for ASD [93,94]. Very recently, the efficacy/tolerability of IVIG infusion has been demonstrated in ASD children with immune dysfunction [95]. Fourteen ASD children were treated with IVIG at a dose of 1 g/kg for ten 21-day treatment cycles. Standardized cognitive and behavioral tests indicated significant improvements together with significant decrease in biomarkers of inflammation [95]. The effectiveness in the use of IVIG in ASD was first demonstrated in an early open-label study involving ten autistic children with immunodeficiencies treated with IVIG (400 mg/kg/4 weeks for 6 months to 18 months) who showed significant improvements in behavioral issues, eye contact, and social interactions [96]. The low rate of positive responses, as well as expense of the immunologic evaluations and of the IVIG were highlighted in a pioneering study [97]. Corticosteroid therapy has also been proposed in ASD management. A single case reported improvement in speech and behavior after oral prednisolone therapy in an autistic child with autoimmune lymphoproliferative syndrome [98]. Corticosteroid therapy has shown positive effects in regressive autism with improvements in language and behavior in young autistic children (aged 3–5 years) [99].

## 5. Conclusions

Neuro-inflammation and neuro-immune abnormalities have now been established in ASD development and maintenance. These alterations can be considered as pathophysiological processes typical of ASD and not only co-morbidities. Also, several ASD-associated symptoms (i.e., seizures, sleep disturbances and gastrointestinal problems) could be influenced by altered immune functions [100].

Several cell types are in charge of driving these harmful bioprocesses. Above all, monocytes/macrophages/microglia cells (the myeloid lineage) are more committed to molecular pro-inflammatory changes. These cells could be considered as cellular markers or as source of potential biomarkers in order to better identify neuro-inflammatory components of ASD. Future research could address possible cytokine biomarkers for ASD [101], in order to establish not only treatment options, but also useful measures of response to treatment [102]. Further, as neuro-inflammation contributes to a significant subset of ASD [103], specific identification of ASD endophenotypes, likely through an integrate approach with the characterization of inflammatory biomarkers, immune subtypes of cells involved and gastrointestinal status, could be useful for personalized approaches. 

Addressing inflammatory processes could be the aim of the next pharmacological therapy for ASD. Future therapies could also address the changes in neuro-glial responses in the brain and restore neuro-immune alterations. Measurements of immune-related biomarkers before and after a putative treatment could help in understanding the effectiveness of the therapeutic intervention.

Therapeutic intervention by restoring gut homeostasis in ASD has already been proposed by the use of probiotics [30,104]. Vitamin A treatment has proven efficacy in restoring gut homeostasis in ASD children, probably by upregulating the Bacteroidetes/Firmicutes ratio [105].

Some limitations should be considered before claiming definitive progress toward effective treatment. It remains to be determined if pro-inflammatory cytokine levels change over time, and frequent analysis should be done in a time-course setting to discover this. Also, differences in the several tests used for ASD diagnosis could influence the samples sizes that need to be analyzed to determine treatment effectiveness; however, most common tests tend to be normalized. Another issue that needs to be addressed in progress toward effective treatment would be the statistical correction used for multiple comparisons, as well as the sample sources (whole blood, serum, plasma, PBMCs, see Table 1 and Table 2) and the biomolecules examined (protein or mRNA). These limitations need to be addressed in further detailed studies.

## Figures and Tables

**Table 1:** Cytokine profiles examined as per the reference articles. Arrows indicate over- or under-production; equal sign indicates no changes. Third column indicates the sample sources. Note that some cytokines (TNF-α; IL-1β, IL-12p40, IL-12p70, IL-17) show increased over-production in all (or almost all) the studies examined. Some limitations should be considered where there is disagreement: sample sources, changes in cytokine profiles over time, and statistical corrections.

Reference	Altered Cytokines	Notes
[14]	IL-1β; IL-6; IL-12p40; IL-8 }↑ IL-2; IL-4; IL-5; IL-6; IL-10; IL-13; IFN-γ; TNF-α } =	plasma
[16]	IL-2; IL-4; IL-13; IFN-γ; IL-5 }↑ IL-10 } =	PBMC
[17]	IL-1β; IL-6; IL-17; IL-12p40; IL-12p70 }↑	plasma
[18]	TNF-α; IL-1β; IL-17 }↑	serum mRNA
[19]	Eotaxin; TGF-β; TNF-α }↑ IL-8 } =	plasma
[20]	TNF-α; IL-6; IL-17 }↑ IL-2 }↓ IL-4; TGF-β } =	whole blood mRNA
[21]	S100B; TNF- α }↑ IL-1β; IFN-γ; IL-6 } =	plasma
[22]	TNF-α; IL-6; IL-1 }↑	serum
[23]	IL-1β; IL-1βa; IL-12p70; IL-8; IL-5; IL-13; IL-17 }↑	plasma

**Table 2:** Cytokine profiles examined by cytokine type. Arrows indicate over- or under-production; equal sign indicates no changes. The third column indicates sample sources. The fourth column indicates the appropriate reference. Interestingly, some cytokines show the same increased levels (pro-inflammatory IL-12p40, IL-12p70, IL-17) or no changes (anti-inflammatory IL-10) in all the studies examined; increased levels of pro-inflammatory IL-1β and TNF-α were found in almost all cases. Other cytokine levels are different among studies, likely reflecting some limitations: sample sources, changes in cytokine profiles over time, and statistical corrections.

Cytokine	Alteration	Sample	Reference
IL-1β	↑ ↑ ↑ =	Plasma Serum (mRNA) Serum Plasma	[14,17,23] [18] [22] [21]
IL-2	= ↑ ↓	Plasma PBMC Whole blood (mRNA)	[14] [16] [20]
IL-4	= ↑ =	Plasma PBMC Whole blood (mRNA)	[14] [16] [20]
IL-5	= ↑ ↑	Plasma PBMC Plasma	[14] [16] [23]
IL-6	= ↑ ↑ = ↑	Plasma Plasma Whole blood (mRNA) Plasma Serum	[14] [17] [20] [21] [22]
IL-8	↑ =	Plasma Plasma	[14,23] [19]
IL-10	= =	Plasma PBMC	[14] [16]
IL-12p40	↑	Plasma	[14,17]
IL-12p70	↑	Plasma	[17,23]
IL-13	= ↑ ↑	Plasma PBMC Plasma	[14] [16] [23]
IL-17	↑ ↑ ↑	Plasma Serum (mRNA) Whole blood (mRNA)	[17,23] [18] [20]
Eotaxin	↑	Plasma	[19]
S-100b	↑	Plasma	[21]
TGF-β	↑ =	Plasma Whole blood (mRNA)	[19] [20]
TNF-α	= ↑ ↑ ↑ ↑	Plasma Serum (mRNA) Plasma Whole blood (mRNA) Serum	[14] [18] [19,21] [20] [22]
IFN-γ	= ↑	Plasma PBMC	[14,21] [16]

**Table 3:** Cytokine profiles examined by separate cytokine type in the ASD brain (referring to Section 3). Arrows indicate over-production. Third column indicates sample sources. Fourth column indicates the appropriate reference. Interestingly, brain pro-inflammatory cytokine over-production is parallel to what found in periphery (Table 2).

Cytokine	Alteration	Sample	Reference
IL-1β	↑	Brain	[45]
IL-6	↑	Brain	[41,42,43,45]
IL-17	↑	Brain	[45]
TNF-α	↑	Brain	[45]
